# Outstanding Reviewers for *Chemical Science* in 2017

**DOI:** 10.1039/c8sc90078g

**Published:** 2018-05-01

## Abstract

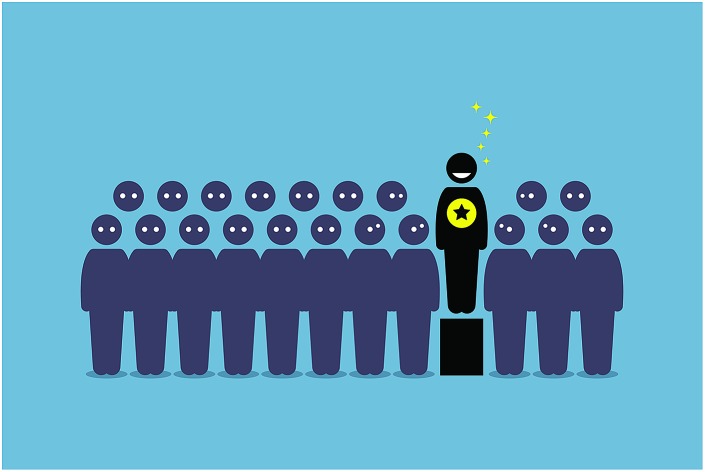
We would like to take this opportunity to highlight the Outstanding Reviewers for *Chemical Science* in 2017, as selected by the editorial team for their significant contribution to the journal.

## 

We would like to take this opportunity to thank all of *Chemical Science*’s reviewers, and in particular highlight the Outstanding Reviewers for the journal in 2017, as selected by the editorial team for their significant contribution to *Chemical Science*. We announce our Outstanding Reviewers annually and each receives a certificate to give recognition for their contribution. The reviewers have been chosen based on the number, timeliness and quality of the reports completed over the last 12 months.

Professor Dr Lutz Ackermann

Georg-August-Universitaet

ORCID: 0000-0001-7034-8772

Professor Mircea Dinca

MIT

ORCID: 0000-0002-1262-1264

Professor Dr Frank Glorius

University of Muenster

Dr Takashi Hisatomi

The University of Tokyo

ORCID: 0000-0002-5009-2383

Professor Rei Kinjo

Nanyang Technological University

ORCID: 0000-0002-4425-3937

Professor Jun Kubota

Fukuoka University

Professor Akihiko Kudo

Tokyo University of Science

Professor Dr Armido Studer

WWU Muenster

ORCID: 0000-0002-1706-513X

Professor Bo Tang

Shangdong Normal University

ORCID: 0000-0002-8712-7025

Dr Jay Winkler

California Institute of Technology

We would also like to thank the *Chemical Science* Editorial and Advisory Boards and the chemical sciences community for their continued support of the journal, as authors, reviewers and readers.

May Copsey, Executive Editor

Susan Weatherby, Editorial Production Manager

